# Rural-urban mortality inequalities in four Nordic welfare states

**DOI:** 10.1177/1403494820921684

**Published:** 2020-05-26

**Authors:** Sven Bremberg

**Affiliations:** Department of Global Public Health, Karolinska Institutet, Stockholm, Sweden

**Keywords:** mortality, Nordic, rural, urban

## Abstract

In the Nordic countries, there are ambitious welfare policies that might reduce rural-urban health disparities. Aim: To investigate the effect of population density on health in four Nordic countries. Methods: The health outcomes analysed were life expectancy and potential years of life lost. The effect of population density was appraised as the difference in life expectancy/ potential years of life lost by a 10-fold increase of population density. Results: In Finland, Norway and Sweden, mortality rates were consistently higher in less densely populated municipalities. These disparities increased over time. ***Conclusions*: The welfare efforts to offset rural-urban disparities have mostly not been sufficient**.

## Introduction

In all Nordic countries, there are policies that aim to reduce health inequalities. These policies specifically emphasise social inequalities. Geographical disparities, including rural-urban differences, are also covered in the relevant governmental documents [1, 2]. Due to the inequality health policies, the geographical health differences might be expected to have levelled out in the Nordic countries, such that differences between rural and urban areas, as reflected in mortality rates depending on population density (PD), have been reduced.

A difference in health between rural and urban areas might be linked to specific risk factors. Incomes are usually lower in rural than in urban areas [3] and it is well known that mortality risks are higher for individuals with low incomes [4]. Therefore, lower incomes in areas with low PD might be one determinant for inferior health in these areas. In rural areas there are fewer opportunities for employment with high incomes. Accordingly, it is justified to understand that the level of income is a mediator between PD and the level of health. Inequality policies might be expected to reduce such differences. The same is true of the situation that applies to the level of education. Educational levels are usually lower in rural populations [3] and a low level of education accompanies worse health [5]. Policies can affect this situation.

The aim of the study was to investigate if rural-urban differences, reflected in municipal PD, affect mortality rates in four Nordic welfare states.

## Methods

The units of study were municipalities in Denmark, Finland, Norway and Sweden. Mortality rates were assessed, either directly or as calculations of life expectancy at birth (LE) or the number of potential years of life lost (PYLL) at ages 0–80 years per 100,000 inhabitants. Municipal data on PD, mortality rates, LE and PYLL were obtained from national sources: in Denmark from Statistics Denmark, in Finland from the Finnish National Institute for Health and Welfare, in Norway from Folkhelseinstituttet and in Sweden from Statistics Sweden.

In the analyses, the LE/PYLL rate was linearly regressed with log10 for PD (inhabitants per square kilometres). The explanatory values of the regression equation were assessed as the unadjusted R^2^ value. The effect of PD was assessed as the difference in LE/PYLL rate for a 10-fold increase of PD. The difference was presented as Cohen’s *d*, that is, as difference/SD. All analyses were performed in MS Excel.

## Results

Data on PD and LE/PYLL were available from Denmark, Finland, Norway and Sweden for 94/98, 183/311, 175/423 and 290/290 municipalities, respectively. Municipalities with small populations had been excluded because the estimates on LE/PYLL in these municipalities were considered to be unreliable.

In Denmark, there was only a 50-fold municipal variation of PD whereas in Finland, Norway and Sweden a 7000-fold, a 1000-fold and a 10,000-fold variation, respectively, were found, see Table I. Thus, Denmark differed from other countries.

**Table I.** Effect of municipal PD on LE at birth or PYLL per 100,000 in females and males in Denmark, Finland, Norway and Sweden.

Country, period, sex, municipal PD median inhabitants/km^2^ (range)	LE years mean (SD)	PYLL per 100,000 mean (SD)	Explanatory value of the regression equation (R^2^)	Effect size (Cohen’s *d*) of 10 times increase of PD
Denmark, 2014–2018, both sexes, PD 44 (12.6–613)	80.8 (0.994)	-	0.00424	-0.242 (ns)
Finland, 1990, both sexes, PD 18.2 (0.4–2826)	-	10275 (1698)	0.160	0.633 (*p*<0.001)
Finland, 2017, both sexes, PD 18.2 (0.4–2826)	-	6439 (1380)	0.117	0.749 (*p*<0.001)
Norway, 2003–2017, females, PD, 33 (2–1818)	83.4 (0.973)	-	0.023	0.237 (*p*<0.05)
Norway 2003–2017 males, PD, 33 (2–1818)	79 (1.09)	-	0.106	0.516 (*p*<0.001)
Sweden, 1998–2002, females, PD 26.4 (0.3–3997)	81.9 (1.05)	-	0.104	0.451 (*p*<0.001)
Sweden, 1998–2002, males, PD 26.4 (0.3–3997)	77.3 (1.23)	-	0.150	0.546 (*p*<0.001)
Sweden, 2013–2017, females, PD 27.5 (0.2–5308)	83.7 (1.04)	-	0.172	0.565 (*p*<0.001)
Sweden, 2013–2017, males, PD 27.5 (0.2–5308)	80.1 (1.39)	-	0.222	0.641 (*p*<0.001)

PD: population density; LE: life expectancy; PYLL: potential years of life lost.

In all countries except Denmark, mortality rates decreased with increasing PD. In Norway and Sweden, a 10-fold increase of PD resulted in an increase of LE that corresponded to 0.237–0.641 SD, which meant 0.231–0.891 years. The largest effect was found in Swedish males in 2013–2017 where a 10,000-fold difference in PD corresponded to an LE difference of 3.6 years.

In Finland, mortality rates were presented as PYLL. The effect of a 10-fold increase varied by 0.633–0.749 SD, namely, the effect was similar to the effects on LE in Norway and Sweden.

The effects of PD were consistently larger in males than in females. The trend over time was also studied in Finland and Sweden. The effect of PD on health outcomes increased over time in both countries.

The effect of municipal PD on male mortality rates was studied in Sweden in 1989–2018 in five age groups, 0–4, 15–24, 35–44, 55–64 and 75–84 years (data not shown). An increase in PD resulted in reduced mortality rates in all age groups. At 0–4 years of age, a 10-fold increase of PD resulted in a 0.459 SD reduction of mortality whereas at 75–84 years of age the reduction was slightly larger, 0.708 SD.

## Discussion

The study demonstrates that in Finland, Norway and Sweden, high mortality rates at the municipal level were associated with low PD. Denmark forms an exception with no differences between municipalities with respect to PD. A potential explanation might a narrower range of municipal PD in Denmark, compared to the other Nordic countries.

In both Finland and Sweden, the effect of PD on health increased over time. A potential explanation might be an ongoing shift in high-income countries towards consumption and production of services instead of production of commodities and industrial goods. This shift towards services benefits people living in areas with high PD.

In Finland, Norway and Sweden, male health was more affected by PD than female health [6–8].

In Finland, Norway and Sweden, mortality rates were consistently higher in less densely populated municipalities. The welfare efforts to offset this gap have obviously not been sufficient. That is not unexpected when the two potential mechanisms are considered – less opportunity for high-income employment and less access to tertiary education in rural areas.

The study builds on official municipal mortality rates, either assessed directly or through calculating LE or PYLL. Data from the countries in total were considered, except for municipalities with small populations. Thus, the findings were reasonably representative and reliable.

The assessments of PD were done with the municipality as a unit. Within a municipality, people are not evenly settled. Instead, they are often concentrated in a few places with relatively high PD. This limitation has probably underestimated the effect of PD on health.
